# Dual RONS-responsive chemiluminescence-guided multimodal thrombolysis based on an aggregation-induced emission cobalt-porphyrin nanoplatform

**DOI:** 10.1039/d6sc02507b

**Published:** 2026-05-13

**Authors:** Ziwei Wang, Liping Zhang, Zihan Wu, Kaijia Lin, Yachao Wang, Gelin Xu, Feng Chi, Weijin Zhu, Qianwen Liu, Dongxia Zhu, Martin R. Bryce, Ben Zhong Tang, Lijie Ren

**Affiliations:** a Department of Neurology, Inst Translat Med, The First Affiliated Hospital of Shenzhen University, Shenzhen Second People's Hospital Shenzhen 518035 China zhanglp914@nenu.edu.cn rlj2020@email.szu.edu.cn; b Key Laboratory of Nanobiosensing and Nanobioanalysis at Universities of Jilin Province, Department of Chemistry, Northeast Normal University 5268 Renmin Street Changchun Jilin Province 130024 China zhudx047@nenu.edu.cn; c Department of Chemistry, Durham University Durham DH1 3LE UK m.r.bryce@durham.ac.uk; d School of Science and Engineering, Shenzhen Institute of Aggregate Science and Technology, The Chinese University of Hong Kong Shenzhen Guangdong 518172 China tangbenz@cuhk.edu.cn

## Abstract

Carotid artery thrombosis carries a high risk of disability and mortality. Organic small molecules can be used to create personalized thrombosis theranostics in precision medicine. However, due to poor water solubility, instability, and competition in energy transfer mechanisms involved in imaging and treatment, the development of small-molecule probes with efficient therapeutic performance remains challenging. Herein, we report the first bimetallic, deep-penetrating aggregation-induced emission (AIE) cobalt-porphyrin-based nanoparticles (Co2Ir NPs) that enable the simultaneous “precise targeting, accurate treatment, and effective repair” of thrombosis. Co2Ir NPs undergo dual response to reactive oxygen and nitrogen species (RONS), releasing Co2Ir, which reacts with endogenous ONOO^−^ for chemiluminescence imaging, thereby allowing precise location of a thrombus. Co2Ir generates ^1^O_2_ and heat *via* photodynamic and photothermal therapy (PDT/PTT) under laser irradiation to dissolve thrombus surfaces, while sonodynamic therapy (SDT) drives the NPs into the thrombi to 8 mm penetration depth, with 82% thrombolysis efficiency without secondary embolism. Concurrently, Co2Ir NPs promote vascular reconstruction by upregulating HO-1 expression to exert antioxidant effects, and by downregulating pro-inflammatory factors like TNF-α and IL-6; this dual effect restores the thrombus site to normal. With its exceptional thrombolytic penetration and potent anti-inflammatory effects, this stimulus-responsive chemiluminescent probe holds great promise for the precise visualization and targeted treatment of thrombi.

## Abstract 2

A cobalt-porphyrin nanoplatform with chemiluminescence imaging for multimodal thrombolysis is reported. Co2Ir NPs exert antioxidant effects and prevent secondary embolism.

## Graphical abstract

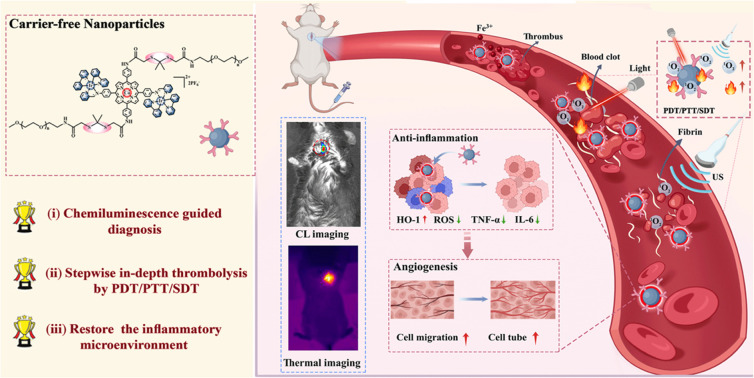

## Introduction

Carotid artery thrombosis, particularly in the internal carotid artery, has a high risk of disability and mortality, due to its complex and multifaceted characteristics.^1–4^ The current standard treatments include mechanical thrombectomy (*e.g.*, stent retrievers) and drug thrombolysis (*e.g.*, tissue plasminogen activators).^5,6^ However, both these treatments have limitations: mechanical thrombectomy may damage endothelial cells, causing restenosis or distal occlusion; thrombolytic drugs have poor efficacy for deep thrombosis due to their short half-life and poor targeting, and high doses can cause bleeding side effects.^7–10^ Fortunately, nanotechnology-based non-invasive thrombosis treatments are emerging, offering greater safety by minimizing the risks of damage to healthy tissue.^11,12^ Meanwhile, the integration of contrast agents into nanoscale platforms can achieve precise diagnosis and treatment of thrombosis.^13,14^ However, the existing diagnostic tools mainly focus on the static morphological assessment of thrombosis, and they are unable to capture the dynamic changes during thrombosis formation, especially for inflammatory responses.^15,16^ The information gap between diagnosis and treatment results in a lack of real-time data to support the treatments in clinical practice.^17,18^ At the same time, synthetically endowing a single-molecule nanoplatform to enable precise vascular diagnostics and multifunctional therapeutic synergy is a daunting task that is still in its infancy.

Technologies based on photothermal and photodynamic therapies (PTT/PDT) have significant advantages in thrombus treatments due to their controllability and non-invasiveness.^19–21^ Unfortunately, their efficacy is limited by low tissue penetration depth, restricting their action primarily to thrombus surfaces, with the inability to treat deep-seated thrombi effectively.^22–26^ In contrast, sonodynamic therapy (SDT) using low-intensity focused ultrasound (US) offers deeper tissue penetration with precise localization and minimal damage to surrounding healthy tissues.^27,28^ US can promote thrombus fragmentation through cavitation effects and mechanical vibrations while also enhancing drug delivery or activating deep photosensitizers (PSs).^29,30^ The current organic small-molecule sensitizers (such as porphyrins and phthalocyanines) have significant limitations: the large π-conjugated structure easily results in aggregation-caused quenching (ACQ) of the excited state, which, in turn, leads to poor generation of the reactive oxygen species (ROS) needed for therapeutic applications.^31–36^ At present, molecular sensitizers that can synergistically achieve phototherapy and sonotherapy are in the early exploration stage due to their strict structural requirements.^37^ Therefore, obtaining an effective photoacoustic response through molecular design is highly challenging.

Vascular embolism triggers local changes, characterized by excessive accumulation of ROS, leading to persistent deterioration of the thrombotic inflammatory microenvironment.^38–40^ This effect exacerbates endothelial cell damage and promotes thrombus proliferation and vascular occlusion; however, these side effects have been largely overlooked in prior studies.^41–43^ Therefore, precise regulation of the inflammatory microenvironment and endothelial repair post-thrombosis has become critical for enhancing the efficacy of anti-thrombotic therapies.^44^ Metal elements or complexes incorporating metals in the d-block of the periodic table, such as transition metal-based smart nanosystems (TMSNs), exhibit enhanced electron transfer ability, strong electrocatalytic activity, and high photothermal conversion efficiency due to the specific lattice structure of metals.^45^ TMSNs as nanozymes have been applied to treat inflammatory diseases caused by oxidative stress due to their ability to mimic enzyme activity.^46^ For example, cobalt protoporphyrin (CoPP) induces heme oxygenase-1 (HO-1) to exert multiple beneficial effects, including reducing oxidative stress, suppressing inflammation, and inhibiting apoptosis.^47–49^ However, the clinical application of CoPP is limited by dose-dependent factors as well as the cytotoxicity resulting from excessive exposure.^50^ Therefore, the design of a responsive metal complex with targeted delivery for *in situ* self-assembly and on-demand release is crucial for controlling the selective and safe delivery of drugs in the dynamic *in vivo* environment.

The goal of thrombolytic therapy is to achieve personalized diagnosis and treatment based on individual characteristics.^51–53^ Fluorescence imaging enables the visualization of dynamic biological processes at the molecular level.^25,54,55^ However, the techniques are limited by tissue scattering and autofluorescence, reducing imaging sensitivity in deep tissues.^56–59^ In response to the above problems, chemiluminescence (CL) imaging has unique advantages. The process generates photons by chemical reactions rather than by external excitation, which effectively avoids interference from tissue autofluorescence, thereby achieving higher imaging sensitivity.^60–63^ Hence, CL technology is potentially more suitable for *in vivo* deep-tissue imaging.^64,65^ Conventional “always-on” fluorescent probes rely on concentration differences to detect biomarkers, which results in insufficient specificity and the inability to distinguish between normal tissues and lesion sites.^66^ In contrast, activatable CL probes employ an “off-on” sensing mechanism, producing signals only upon recognition of target biomarkers, thereby achieving a higher signal-to-background ratio (SBR) and lower detection limits.^67–69^ However, there is a current lack of clear design principles for high-performance CL materials. Therefore, how to directly transform traditional CL materials into CL probes with ideal properties (high penetration depth and long half-life *t*_1/2_) through molecular engineering, while achieving “activatable” responses to specific biomarkers for integrated diagnosis and treatment, urgently needs to be addressed.

Although molecules with the tetrapyrrole skeleton (*e.g.*, porphyrins, phthalocyanines) dominate clinically approved PDT sensitizers, they can also have limitations, such as high lipophilicity, easy aggregation, photoinstability and hepatotoxicity.^70,71^ Iridium-based composite PSs have a d^6^ electronic configuration and tend to adopt an octahedral structure, which imparts a high degree of tunability.^72–75^ Additionally, the versatile redox and photophysical properties of the iridium center facilitate integrated diagnosis and treatment,^76–79^ although the clinical application of iridium PSs can be hindered by poor solubility and limited targeting efficiency.^80–83^ Conventional carrier-based delivery systems often suffer from low drug loading and significant batch-to-batch variability.^84,85^ Therefore, there is a focus on small-molecule nanoparticles with well-defined chemical structures, high synthetic reproducibility and good metabolism, which can achieve high and precise PS loading efficiency.

In the light of the above considerations, we now report for the first time deep-penetrating bimetallic aggregation-induced emission (AIE) nanoparticles, named Co2Ir NPs, as a single-component platform that can realize four key characteristics simultaneously in practical applications: (i) CL imaging-guided diagnosis of thrombi; (ii) in-depth thrombolysis achieved by combined PDT/PTT/SDT; (iii) the recovery of antioxidant activity at the thrombus inflammatory microenvironment; and (iv) the carrier-free nanoparticles have dual response to reactive oxygen and nitrogen species (RONS) to ensure the safety and accuracy of *in vivo* therapy. Our molecular design utilizes cobalt-porphyrin as a bridge to flexibly connect two cationic cyclometallated Ir(C^N)_2_(N^N)^+^ complexes to construct Co2Ir. Polyethylene glycol chains incorporating RONS-sensitive thioketal (TK) bonds serve as a degradable hydrophilic outer layer, while the Co2Ir core is hydrophobic, forming water-soluble, activatable Co2Ir NPs.

**Scheme 1:**
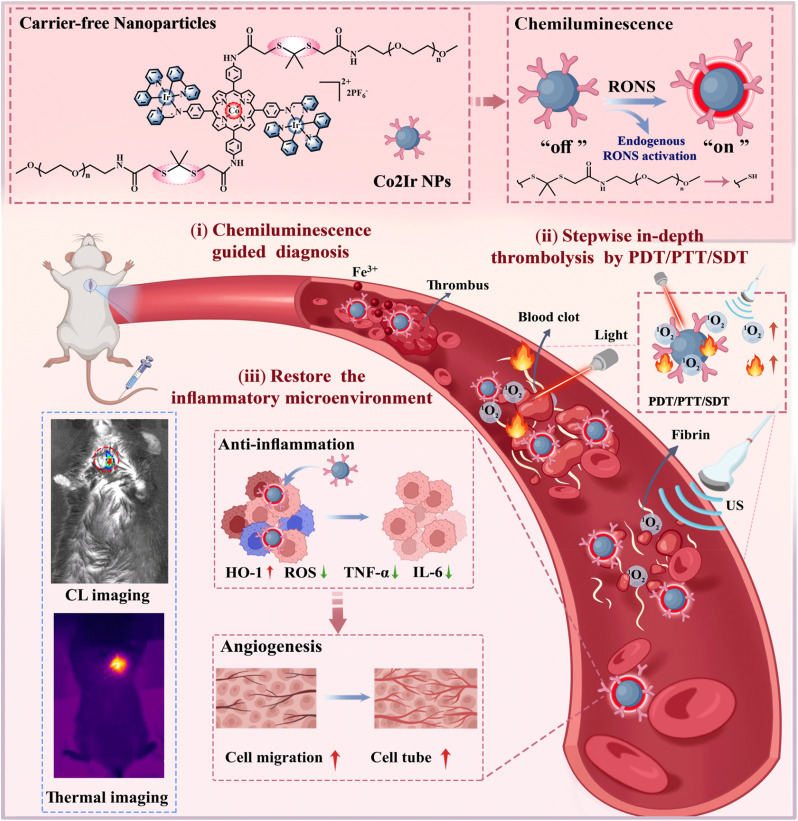
Schematic illustration of thrombotic therapy with Co2Ir NPs.

These NPs function as a single-component platform integrating CL-guided PDT/PTT/SDT for thrombolysis with excellent anti-inflammatory properties, representing a novel theranostic thrombolytic material. Co2Ir NPs, with RONS-responsive TK bonds, release Co2Ir upon exposure to the high H_2_O_2_ concentration of the thrombus microenvironment. The released Co2Ir then reacts with another endogenous RONS, namely ONOO^−^, to generate CL for precise localization. Under laser irradiation, the AIE properties of Co2Ir effectively reduce non-radiative transition pathways by restricting intramolecular motion, thereby inhibiting the quenching of triplet excitons, to achieve enhanced ^1^O_2_ production and heat generation for initial dissolution during PDT. Meanwhile, SDT promotes the deep reperfusion of the NPs. Post-recanalization, Co2Ir upregulates HO-1 (yielding the antioxidant bilirubin) and downregulates tumor necrosis factor (TNF-α) and pro-inflammatory cytokine (IL-6), balancing redox and inflammation to restore vascular function. This theranostic nanoplatform enables depth-precise thrombolysis and functional recovery *via* RONS activation (Scheme 1).

## Results and discussion

**Fig. 1:**
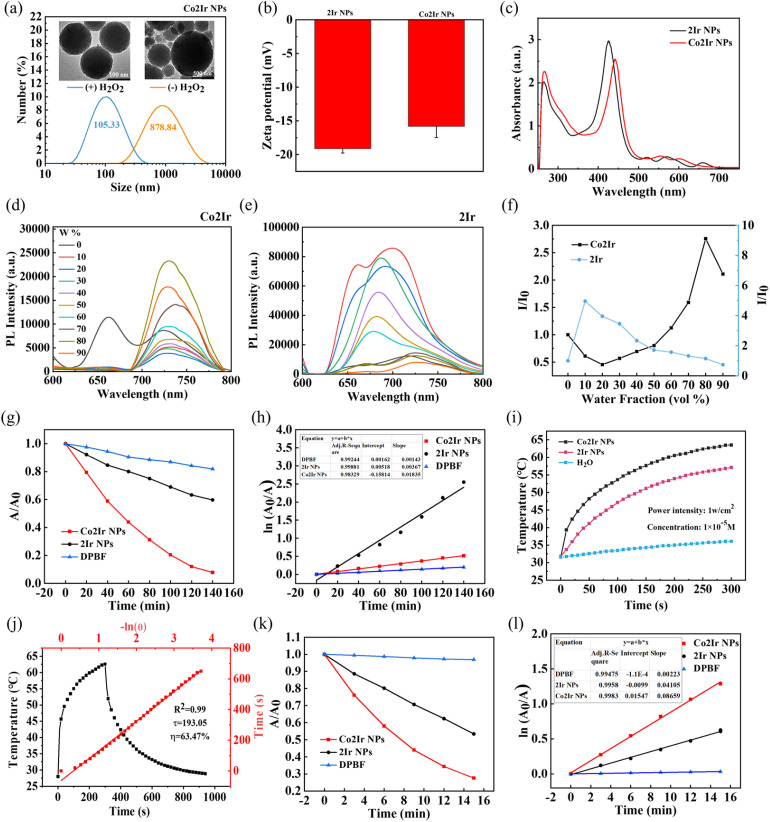
(a) Size distribution and TEM image of Co2Ir NPs before (−) and after (+) treatment with H_2_O_2_ by DLS. (b) Zeta potential of 2Ir NPs and Co2Ir NPs. (c) UV-visible absorption spectra of 2Ir NPs and Co2Ir NPs in water. Fluorescence (FL) spectra of Co2Ir (d) and 2Ir (e) in DMSO/water mixtures with different water fractions (0–90 vol%). (f) FL intensities at 730 nm of Co2Ir and 2Ir in a mixed solvent with various DMSO/H_2_O ratios (*I*/*I*_0_). *I*_0_ and *I* are respectively the maximum FL intensities of Co2Ir and 2Ir in pure DMSO and DMSO/H_2_O mixtures. (g) Comparison of decay rates of 2Ir NPs and Co2Ir NPs under irradiation (635 nm, 1 W cm^−2^); *A*_0_ = DPBF absorption under no irradiation, *A* = real-time absorption of DPBF at different irradiation times. (h) ^1^O_2_ generation kinetics over time. (i) Photothermal profiles of H_2_O, 2Ir NPs and Co2Ir NPs under 635 nm laser irradiation. (j) Plot of cooling time *versus* negative natural logarithm of the temperature obtained from the cooling stage of Co2Ir NPs. (k) UV-visible absorption spectral changes of DPBF at 410 nm in the presence of 2Ir NPs and Co2Ir NPs under ultrasound irradiation (1.0 W cm^−2^, 1 MHz, 50% duty cycle). (l) ^1^O_2_ generation kinetics for DPBF, 2Ir NPs and Co2Ir NPs over time.

A hydrophobic cobalt-porphyrin core linked to two cyclometallated Ir(C^N)_2_(N^N)^+^ complexes (Co2Ir) was first synthesized (Scheme S1 and Fig. S1–S4). Subsequently, a simple amide condensation reaction was used to conjugate Co2Ir with a RONS-sensitive polymeric carrier containing TK bonds, thereby obtaining the water-soluble Co2Ir nanoparticles (Co2Ir NPs). 2Ir NPs, without Co^2+^ moieties, were synthesized from 2Ir for comparison purposes. The structure of the RONS-sensitive organic precursor was validated using FT-IR spectra (Fig. S5). The successful synthesis of Co2Ir was also evidenced by the disappearance of the N–H bending vibrational peak associated with the porphyrin ring at 963 cm^−1^, and the appearance of the N–Co metal-sensitive peak at 1006 cm^−1^. The C=O peak at 1720 cm^−1^ and the peak that appeared at 2870 cm^−1^, corresponding to the stretching vibration of the saturated carbon–hydrogen bond (C–H), indicate the successful preparation of the nanoparticles. The Co2Ir NPs and 2Ir NPs were analyzed by transmission electron microscopy (TEM) and dynamic light scattering (DLS). The NPs were circular with particle sizes of 105.33 and 90.41 nm, respectively. In the presence of 1 mM H_2_O_2_, the Co2Ir NPs and 2Ir NPs disintegrated over time and completely disassembled, resulting in a rapid increase in the particle size (Fig. 1a and S6), which indicated the cleavage of the TK moieties triggered by H_2_O_2_. The zeta potentials of 2Ir NPs and Co2Ir NPs are −19.1 and −15.8 mV, respectively, which is conducive to achieving excellent serum stability (Fig. 1b). As shown in Fig. 1d, Co2Ir was soluble in pure DMSO solution, with weak emission at 730 nm, which significantly increased in intensity when the water fraction reached 80%. At 90% water fraction, the emission intensity of Co2Ir was still ∼2 times higher than at 0% (Fig. 1f), indicating that Co2Ir is a typical AIE material.

In contrast, 2Ir does not show AIE (Fig. 1e). *In vivo* optical imaging must contend with the limitations imposed by the optical window of tissue (600–1000 nm).^86^ The addition of Co ions enhanced the absorption of Co2Ir NPs at 635 nm, as compared to 2Ir NPs (Fig. 1c). This is beneficial for phototherapy using a 635 nm laser. Consequently, the absorbance of the probe 1,3-diphenylisobenzofuran (DPBF) under 635 nm irradiation was monitored by UV-visible absorption spectroscopy (Figure S7). The absorbance was significantly reduced in the presence of Co2Ir NPs, which demonstrated their good ^1^O_2_-generating ability under 635 nm irradiation, resulting in the loss of DPBF's extended π-electron system upon reaction with ^1^O_2_. From the decay curves (Fig. 1g) and the first-order kinetics of ^1^O_2_ production (Fig. 1h), the photocatalytic performance of Co2Ir NPs is stronger than that of 2Ir NPs. A steeper slope represents a faster decay rate and increased ability to generate ^1^O_2_ (Fig. 1h).

The photothermal properties of the NPs were assessed under 635 nm irradiation. Impressively, Co2Ir NPs demonstrated robust heat generation, with the temperature increasing to 63.6 °C (Δ*T* = 32.0 °C) under laser irradiation at a power density of 1 W cm^−2^ (Fig. 1i), and this was investigated by infrared thermal imaging (Figure S8). The temperature increase scaled predictably with increasing power and concentration (Figure S9).

Co2Ir NPs maintained a consistent performance through multiple heating–cooling cycles (Figure S9c), further establishing that these NPs possess excellent photostability. The photothermal conversion efficiency of Co2Ir NPs is 63.47%, significantly higher than that of 2Ir NPs of 53.3% (Fig. 1j and S9d). The detailed calculation method for the photothermal conversion efficiency is provided in the SI. The high *η* value observed for Co2Ir NPs arises from the excellent capability of this nanoscale system to efficiently convert absorbed photon energy into thermal energy. The cobalt core effectively promotes the generation of triplet excitons, thereby facilitating the production of ^1^O_2_ and enhancing therapeutic efficacy.

Porphyrin molecules are excellent sonosensitizers;^87^ therefore, we also tested the sonodynamic performance of Co2Ir NPs. As displayed in Fig. 1k and l, the absorption band of DPBF at 410 nm decreased by >70% in the presence of Co2Ir NPs under low-intensity ultrasound irradiation within 15 min, whereas this absorption decreased by only about 40% with 2Ir NPs, showing the more effective ^1^O_2_ generation ability of Co2Ir NPs under ultrasonic irradiation (Fig. S10). These data convincingly demonstrate that Co2Ir NPs with AIE properties exhibit superior sonodynamic, photodynamic and photothermal properties and also possess excellent thermal stability.^88^ Consequently, Co2Ir NPs are anticipated to be very efficient phototheranostic agents in thrombolysis.

**Fig. 2:**
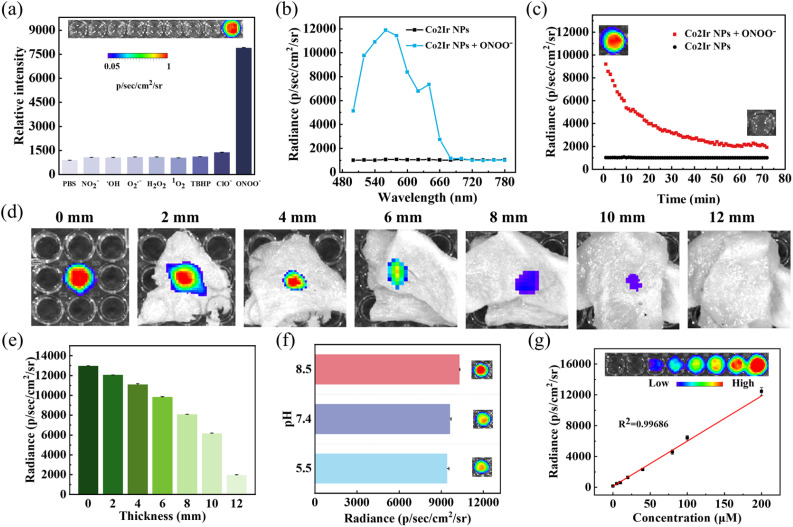
(a) Selectivity of preirradiated Co2Ir NPs towards treatment with different RONS (200 µM) (PBS = phosphate buffered saline; TBHP = *tert*-butyl hydroperoxide). Inset: the CL images acquired by an IVIS imaging system under bioluminescence mode with an open filter. (b) Decay of the persistent luminescence signal of Co2Ir NPs (200 µg mL^−1^) and PBS solution (pH 7.4) over time at room temperature after addition of ONOO^−^ (200 µM). (c) CL spectra of Co2Ir NPs in the absence or presence of ONOO^−^ in PBS solution (pH 7.4). Insets: luminescence images at the beginning and end of the CL of Co2Ir NPs. (d) Tissue penetration depths of the activated CL of preirradiated and ONOO^−^-added Co2Ir NPs with a coverage of chicken breast tissues with different thicknesses. (e) Quantitative analysis of the relative CL intensity in (d). (f) CL spectra of Co2Ir NPs at different pH values. Inset: CL images acquired by an IVIS imaging system under bioluminescence mode with an open filter. (g) Correlation of persistent luminescence intensity of Co2Ir NPs *versus* ONOO^−^ concentration. Inset: the corresponding CL acquired with an IVIS imaging system.

CL, light emission from chemical reactions, is widely used in analytical sensing, bioanalysis, and theranostics.^21^ To achieve specific detection of a thrombus, the CL properties of Co2Ir NPs were tested using various RONS (NO_2_^−^, ˙OH, O_2_˙^−^, H_2_O_2_, ^1^O_2_, TBHP, ClO^−^, ONOO^−^). Among them, Co2Ir NPs oxidized by ONOO^−^ showed the strongest CL signal, 8.7-fold higher than that of the phosphate-buffered saline (PBS) control (Fig. 2a). The CL spectra of Co2Ir NPs were detected at different emission peak positions, and the emission wavelength extended to 680 nm (Fig. 2b). Co2Ir NPs in PBS solution exhibited CL immediately after the addition of ONOO^−^. The CL signal gradually decayed with time and persisted for 70 minutes, with a higher intensity than that of the sample without ONOO^−^ (Fig. 2c). The predicted reaction pathway is oxidation by ONOO^−^ to form an unstable Co2Ir-dioxetane intermediate, which degrades to Co2Ir-aldehyde, releasing photons and CL.^21,60*b*^

The imaging efficiency of drugs *in vivo* depends critically on their tissue penetration depth. Therefore, the CL penetration of Co2Ir NPs through chicken breast tissues of varying thickness was tested. As shown in Fig. 2d and e, CL signals remained detectable even at 10 mm depth. The CL intensity of Co2Ir NPs was hardly affected by changes in the pH in the range 5.5 to 8.5 (Fig. 2f). The CL signals of Co2Ir NPs showed a linear relationship with the concentration of ONOO^−^, and the correlation coefficient reached 0.99686 (Fig. 2g). Therefore, Co2Ir NPs with a strong CL signal have great potential for monitoring deep thrombus changes.

**Fig. 3:**
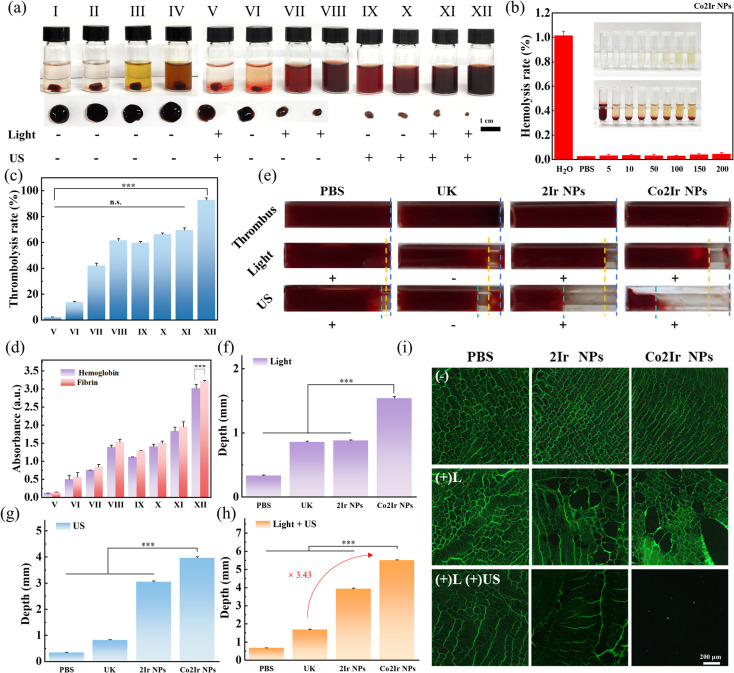
(a) Photographs of the blood clot solution after different treatments (I: PBS, II: UK (urokinase), III: 2Ir NPs, IV: Co2Ir NPs, V: PBS + L (light) + US (ultrasound), VI: UK (–L), VII: 2Ir NPs + L, VIII: Co2Ir NPs + L, IX: 2Ir NPs + US, X: Co2Ir NPs + US, XI: 2Ir NPs + L + US, XII: Co2Ir NPs + L + US). The residual blood clots were taken out and shown below. (b) The hemolysis rate of red blood cells treated with water and different concentrations (µg mL^−1^) of Co2Ir NPs. Inset shows samples in the presence or absence of the red blood cell solution upon different treatments. (c) The quantified clot-dissolution efficiency of different treatment groups (corresponding to (a)) *in vitro*. (d) The absorbance at 450 and 540 nm in different treatment groups (corresponding to (a)). (e) Images of excavating ∼5 mm long and 2 mm diameter thrombi after different treatments. Quantitative analysis of the depth of thrombolysis under light exposure (f), under US (g) and under light exposure and US (h) (corresponding to (e)). (i) CLSM images of fibrin clot after different treatments. Data are presented as mean ± SD (*n* = 3). *p* < 0.05 was considered statistically significant. *p < 0.05, **p < 0.01, ***p < 0.001, ****p < 0.0001.

The thrombolytic effects of various formulations were then investigated by subjecting blood clots to different treatments (Fig. 3a). Red blood cells (RBCs) are the most abundant cells in blood. Initially, the hemolysis rate was measured to assess the blood compatibility of the nanoparticles, which is crucial for thrombolysis. Using PBS and water as negative and positive controls, respectively, Fig. 3b (for Co2Ir NPs) and Figure S11 (for 2Ir NPs) demonstrate that the hemolysis rate remained below 5% even at NP concentrations up to 200 µg mL^−1^, confirming that the NPs do not damage RBCs and exhibit excellent blood compatibility. Then, the blood clots underwent different treatments. Among all groups, only the control group treated with laser and ultrasound (group V) did not show a significant thrombolytic effect. Meanwhile, the thrombolysis rate in the UK group (VI) within the same treatment time was 14.0%, which was much lower than that of the laser-only group (VII) at 41.84%, group (VIII) at 61.44%, the ultrasound-only group (IX) at 59.60% and group (X) at 66.26%. This indicates that using both methods (laser and ultrasound) should have a stronger thrombolytic effect. Indeed, as expected, the thrombolytic rate of Co2Ir NPs was as high as 93.2%, almost completely dissolving the thrombus (Fig. 3c, group XII). Moreover, the thrombolytic rate of 2Ir NPs was also higher than that of the single treatment method, reaching 69.68% (Fig. 3c). It is worth noting that, under the same conditions, the thrombolytic effect of Co2Ir NPs was stronger than that of Ir NPs in any control group. We attribute this effect to the superior photophysical properties of Co2Ir NPs. Also, the amount of hemoglobin and fibrin released into the supernatant during clot lysis presented a similar trend to lysis efficiency (Fig. 3d). To study the deep thrombolytic ability of Co2Ir NPs, an *in vitro* thrombus model allowed the *in situ* observation of the thrombolysis process of disrupting the entire blood clot by combining SDT, PDT and PTT.

Thrombi, approximately 8 mm in length and 2 mm in diameter, were treated with PBS, pure UK, 2Ir NPs and Co2Ir NPs for 30 min (Fig. 3e). As shown in Fig. 3f, under light-only conditions, the maximum thrombolytic depth of Co2Ir NPs was approximately 1.54 mm. However, the depth that US could achieve reached nearly 3.97 mm (Fig. 3g), which is 2.58 times greater than that in the light-only group. Moreover, in the combined treatment group (+light and +US), the thrombolytic depth increased to 5.7 mm, *i.e.* 3.43 times that of the UK group under the same thrombolysis time (Fig. 3h), achieving complete penetration in the thrombolysis model. This indicates that US enhances the depth of thrombolysis (Fig. S12).

Fibrin cross-linking forms a network structure that traps RBCs and platelets, which are important components of blood clots.^15^ To explore the disruptive effect of RONS on the fibronectin skeleton, the amounts of collapsed skeleton and fragments were distinguished by confocal laser scanning microscopy (CLSM) images after incubating fibrin with NPs and light irradiation (Fig. 3i). After light-only treatment, the collapsed condition of the fibrin is not obvious. However, especially for the Co2Ir NPs + L + US group, the fragments of fibrin were almost completely destroyed. This is consistent with Co2Ir NPs producing a large amount of RONS in the combined mode (+L and +US), which further damaged the fibrin main strand and effectively enhanced the thrombolytic effect. These *in vitro* results indicate that Co2Ir NPs possess excellent biocompatibility and outstanding PDT/PTT/SDT properties. The combined use of multiple methods should enhance thrombolytic effects *in vivo*.

**Fig. 4:**
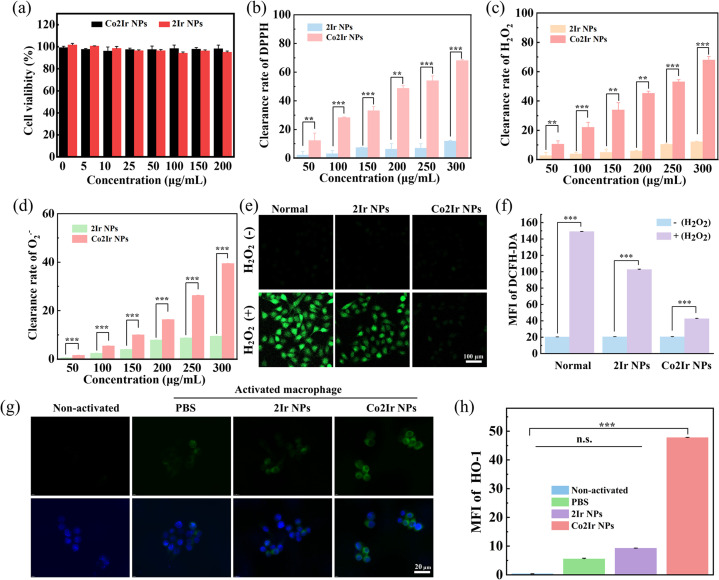
(a) Cell viability was measured by MTT assay after HUVECs were treated with different concentrations of NPs in PBS solvent for 24 h. DPPH (b), H_2_O_2_ (c) and O_2_˙^−^ (d) scavenging activity of 2Ir NPs and Co2Ir NPs. (e) NPs scavenged ROS activity induced by H_2_O_2_-treated HUVECs *in vitro*. (f) Quantitative analysis of the intracellular fluorescence intensity of NPs scavenged RONS activity induced by H_2_O_2_-treated HUVECs *in vitro* (MFI = mean fluorescence intensity). (g) Immunofluorescence staining for HO-1 expression on non-activated and activated RAW264.7 cells after different treatments as indicated. (h) Quantitative analysis of HO-1 FL intensity in (g) (n.s. = not statistically significant). Data are presented as mean ± SD (*n* = 3). *p* < 0.05 was considered statistically significant. *p < 0.05, **p < 0.01, ***p < 0.001, ****p < 0.0001.

Cytotoxicity, or the absence thereof, is critical in assessing the suitability of nanomaterials for biomedical applications. A 3-(4,5-dimethylthiazol-2-yl)-2,5-diphenyltetrazolium bromide (MTT) assay demonstrated that the NPs exhibit no obvious cytotoxicity at a concentration of 200 µg mL^−1^. Cell viability remained as high as 95% when the cells were incubated with varying concentrations (0, 5, 10, 25, 50, 100, 150 and 200 µg mL^−1^ in PBS solvent) in the dark for 24 h (Fig. 4a). The Co^3+^ ions that did not form coordination bonds with porphyrins exhibited significant cytotoxicity and were not suitable for *in vivo* testing (Fig. S13).

These results support the rationality of the Co2Ir NPs structure. The ROS scavenging efficiencies of Co2Ir NPs and 2Ir NPs were evaluated using four representative ROS involved in oxidative stress, namely 2,2-diphenyl-1-(2,4,6-trinitrophenyl)hydrazyl (DPPH), total antioxidant capacity (T-AOC), hydrogen peroxide (H_2_O_2_), and superoxide anion (O_2_˙^−^) (Fig. 4b–d and S14). Both NPs scavenged ROS in a concentration-dependent manner. Notably, Co2Ir NPs, with an increased number of metal cores, exhibited enhanced ROS scavenging compared to 2Ir NPs.

The ability of the NPs to scavenge RONS in human umbilical vein endothelial cells (HUVECs) was investigated. H_2_O_2_ treatment significantly increased ROS levels (green fluorescence) in HUVECs, as detected by 2′,7′-dichlorodihydrofluorescein diacetate (DCFH-DA). Co2Ir NPs exhibited the lowest fluorescence intensity (Fig. 4e), indicating superior ROS-scavenging ability. Quantitative analysis (Fig. 4f) further confirmed that Co2Ir NPs are efficient ROS scavengers. These data indicate that Co2Ir NPs possess significant antioxidant properties. To investigate the antioxidant mechanism of the NPs, we tested the effect of NPs on the production of HO-1, an endogenous antioxidant system that maintains cellular homeostasis under pathological conditions.^89,90^ Intracellular HO-1 levels were assessed using immunofluorescence staining. As presented in Fig. 4g, activated macrophages treated with Co2Ir NPs exhibited 8.54- and 5.26-fold higher HO-1 protein expression compared to those treated with PBS and 2Ir NPs, respectively (Fig. 4h).

**Fig. 5:**
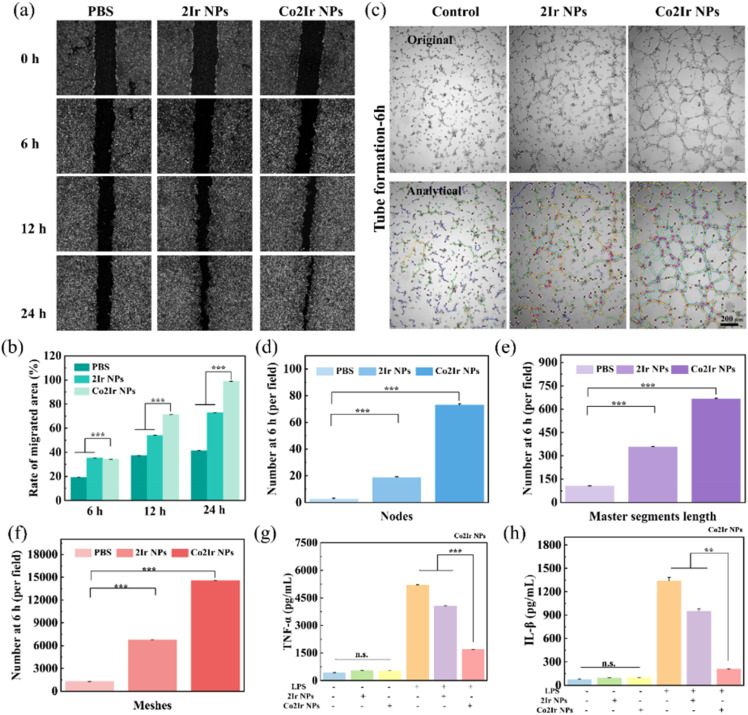
(a) Scratch micrographs of HUVECs grown in media supplemented with PBS, 2Ir NPs or Co2Ir NPs, and (b) corresponding quantitative histograms (*n* = 3). (c) Representative images of tube formation after 6 h incubation of HUVECs with PBS, 2Ir NPs or Co2Ir NPs. Statistics of nodes (d), master segments (e) and meshes (f) formed after 6 h of *in vitro* tube formation experiments for PBS, 2Ir NPs and Co2Ir NPs. Concentrations of (g) TNF-α and (h) IL-6 were determined by an enzyme-linked immunosorbent assay (ELISA) (n.s. = not statistically significant). Data are presented as mean ± SD (*n* = 3). *p* < 0.05 was considered statistically significant. *p < 0.05, **p < 0.01, ***p < 0.001, ****p < 0.0001.

Scratch tests were used to check how the NPs affect cell movement during 24 h of co-incubation (Fig. 5a and b). The PBS group exhibited no significant influence on cell migration. A slight impediment to migration was observed in the 2Ir NPs group. The group treated with Co2Ir NPs displayed improved cell migration. Fig. 5a shows the cell movement in each group. The angiogenic effect of 2Ir NPs and Co2Ir NPs was systematically investigated *in vitro* using a tube formation assay, which is a well-established model for evaluating endothelial cell sprouting and vascular network formation. As documented in Fig. 5c–f, Co2Ir NPs exhibited a remarkable capacity to enhance HUVEC tube formation. Specifically, Co2Ir NPs induced a substantial increase in the formation of intricate vessel-like structures, characterized by a higher density of master junctions (critical branching points) and significantly elongated tube lengths compared to the controls (BPS and 2Ir NPs).

These observations collectively suggest that Co2Ir NPs exert a multifaceted pro-angiogenic effect by facilitating endothelial cell proliferation, promoting directional migration, and stimulating the formation of functional microvascular networks. Such actions are pivotal for enhancing nutrient and oxygen supply to ischemic tissues, highlighting the therapeutic potential of Co2Ir NPs in promoting tissue regeneration and re-epithelialization, particularly in thrombolysis treatment, where revascularization is paramount for restoring tissue viability.

To evaluate the anti-inflammatory properties of Co2Ir NPs, RAW264.7 cells were stimulated with 1 µg mL^−1^ of lipopolysaccharides (LPS) to induce an inflammatory response. As illustrated in Fig. 5g and h, the expression levels of key pro-inflammatory cytokines, tumor necrosis factor-alpha (TNF-α) and interleukin-6 (IL-6), were significantly downregulated in the presence of Co2Ir NPs compared to the control groups. This pronounced reduction in cytokine production highlights the anti-inflammatory activity of Co2Ir NPs in mitigating LPS-induced inflammation. Consequently, Co2Ir NPs, which integrate ROS scavenging and HO-1 upregulation, effectively ameliorated the inflammation and reshaped the thrombus microenvironment, thereby promoting the recovery of blood vessels after thrombolysis.

**Fig. 6:**
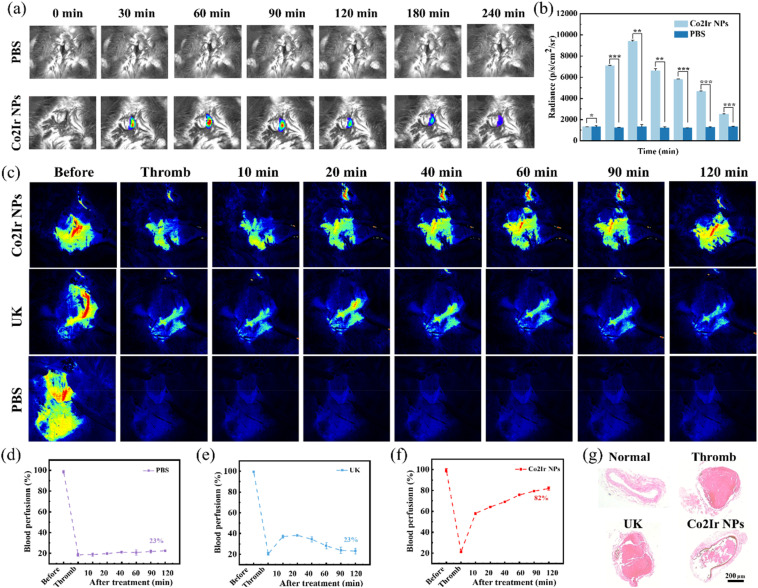
(a) Representative *in vivo* CL images of the thrombotic artery at different times post-injection of PBS and Co2Ir NPs. (b) Quantification of the relative CL intensity of mouse neck thrombi in (a). (c) During laser speckle blood flow monitoring system (LSBFMS) imaging, the right carotid thrombotic artery of each mouse (indicated with black lines) was focused, and its blood perfusion was quantified. Representative LSBFMS images and the corresponding relative blood perfusion of the mouse carotid artery after FeCl_3_ induction and different treatments, namely: PBS (d), free UK (e) and Co2Ir NPs PBS (f). Data are presented as mean ± SD (*n* = 3 mice). (g) H&E staining of blood vessels after different treatments. Scale bar = 200 µm.

We proceeded to investigate the *in vivo* efficacy of Co2Ir NPs for treating obstructive thrombosis. FeCl_3_-induced thrombus-bearing mice intravenously injected with Co2Ir NPs (5 mg kg^−1^) exhibited a significant increase in thrombus-localized CL signal within 60 minutes (Fig. 6a and b). Infrared thermal imaging revealed selective temperature elevation at the thrombus site upon laser irradiation, with minimal changes in surrounding tissue (Fig. S15), enabling real-time tracking *via* CL and photothermal imaging.

In a carotid artery thrombosis model, treatments were compared: PBS (control), free UK (0.04 mg kg^−1^), and Co2Ir NPs + laser/ultrasound (635 nm; 1.0 W cm^−2^, 1 MHz, 50% duty cycle) (Fig. 6c). Laser speckle imaging showed that PBS did not exhibit a significant thrombolytic effect (Fig. 6d). Although free UK partially restored blood flow, blood flow decreased after 60 minutes due to the short half-life of UK (Fig. 6e). In contrast, Co2Ir NPs combined with photodynamic/thermal/sonodynamic therapy (PDT/PTT/SDT) achieved site-specific clot dissolution (Fig. 6f), restoring and stabilizing blood flow without reperfusion-induced bleeding. The thrombolysis rate reached 82% after 120 minutes, with no re-embolism observed. These results highlight that Co2Ir NPs reverse thromboembolism in mice.

Histological analysis of carotid tissues *via* hematoxylin and eosin (H&E) staining (Fig. 6g) revealed distinct differences between the treatment groups. Vessels treated with Co2Ir NPs showed a significantly decreased thrombus area and diminished inflammatory cell infiltration compared to embolized vessels. Notably, treatment with Co2Ir NPs led to histology closer to that of normal vasculature compared with UK treatment. *In vivo* safety evaluation of Co2Ir NPs was conducted *via* injection of PBS, UK, or Co2Ir NPs into mice tail veins. Post-mortem examination of major organs (brain, heart, liver, spleen, lung, and kidney) revealed no significant pathological changes or inflammatory responses in the Co2Ir NPs-treated group (Figure S16). Additionally, comprehensive blood tests confirmed that all hematological parameters remained within normal ranges (Fig. S17). These findings collectively demonstrate the excellent biocompatibility of Co2Ir NPs *in vivo*, a critical prerequisite for safe thrombolytic therapy.

## Conclusions

This study has created a deep-penetrating bimetallic porphyrin nanoplatform (Co2Ir NPs) that integrates diagnostic and therapeutic functions, enabling the simultaneous “precise targeting, accurate treatment, and effective repair” of thrombosis. The Co2Ir NPs locate carotid artery thrombi through H_2_O_2_-responsive bond cleavage at thrombus sites, releasing Co2Ir to react with endogenous ONOO^−^ for CL imaging. The AIE-active Co2Ir generates ^1^O_2_ and heat *via* PDT/PTT under laser irradiation to dissolve thrombus surfaces, while ultrasound drives the NPs *via* SDT deep into thrombi, achieving an 8 mm penetration depth and 82% thrombolysis efficiency without secondary embolism. Concurrently, Co2Ir NPs promote vascular functional reconstruction by upregulating HO-1 expression to exert antioxidant effects and by downregulating pro-inflammatory factors like TNF-α and IL-6. This dual regulatory mechanism of antioxidant and anti-inflammatory effects mitigates oxidative damage and local inflammatory responses, improves the microenvironment, and promotes endothelial repair and restoration of normal vascular function. Stepwise thrombolysis is achieved from surface to depth while facilitating vascular functional reconstruction. Overall, this strategy provides a powerful new approach for synergistic antithrombotic nanomedicine.

## Ethical statement

All animal procedures were performed in accordance with the Guidelines for Care and Use of Laboratory Animals approved by the Animal Ethics Committee of the China Technology Industry Holdings (Shenzhen) Co. Ltd.

## Author contributions

Conceptualization, Z. W. (Ziwei Wang) and D. Z. (Dongxia Zhu); methodology, Z. W. and L. Z. (Liping Zhang); software, Z. W. and Z. H. W. (Zihan Wu); validation, Z. W., L. R. (Lijie Ren) and B. T. (Ben Zhong Tang); formal analysis, Z. W., K. L. (Kaijia Lin), W. Z. (Weijin Zhu) and Q. L. (Qianwen Liu); investigation, Z. W., G. X. (Gelin Xu) and F. C. (Feng Chi); resources, Z. W. and L. Z.; data curation, Z. W. and K. L.; writing—original draft preparation, Z. W. and D. Z.; writing—review and editing, Z. W. and M.R.B. (Martin R. Bryce); project administration, Z. W., Y. W. (Yachao Wang), and K. L.; funding acquisition, D. Z. and M. R. B. All authors have read and agreed to this version of the manuscript.

## Conflicts of interest

There are no conflicts to declare.

## Supplementary Material

SC-017-D6SC02507B-s001

## Data Availability

The data supporting this article are available in the supplementary information (SI). Supplementary information: additional experimental details, characterization data, supplementary figures and tables, and extra mechanism verification related to this study. See DOI: https://doi.org/10.1039/d6sc02507b.
