# Supplementary material for: Dual RONS-responsive chemiluminescence-guided multimodal thrombolysis based on an aggregation-induced emission cobalt-porphyrin nanoplatform
Source: Chem Sci. 2026 May 13;17(24):12120–32. doi: 10.1039/d6sc02507b (PMC13170895; doi:10.1039/d6sc02507b)
Supplement: SC-017-D6SC02507B-s001 [file SC-017-D6SC02507B-s001.pdf]

Supplementary Information (SI) for Chemical Science.
This journal is © The Royal Society of Chemistry 2026

Supporting Information

Dual RONS-Responsive Chemiluminescence-Guided Multimodal Thrombolysis Based on an Aggregation-Induced Emission Cobalt-Porphyrin Nanoplatform

Ziwei Wang,[a,b] Liping Zhang,*[a] Zihan Wu,[b] Kaijia Lin,[a] Yachao Wang,[a] Gelin Xu,[a] Feng Chi,[a] Weijin Zhu,[b] Qianwen Liu,[b] Dongxia Zhu,*[b] Martin R. Bryce,*[c] Ben Zhong Tang*[d] and Lijie Ren*[a]

Experimental Procedures

General Materials and Methods.

Materials for organic synthesis were purchased from Energy Chemical Company. MPEG2000-TK-COOH was purchased from Xi'an ruixi Biological Technology Co., Ltd. 1,3-Diphenylisobenzofuran (DPBF) was purchased from Energy Chemical Company. RPMI Medium 1640 was purchased from Solarbio Life Science Company. Fetal bovine serum (FBS) was purchased from Sigma-Aldrich. 3-(4,5-dimethyl-2-thiazolyl)-2,5-diphenyl-2*H*-tetrazolium bromide (MTT), 7,7'-dichlorofluorescein diacetate (DCFH-DA) and the cell viability (live dead cell staining) assay kit were purchased from Shanghai Beyotime Biotechnology Co. Ltd.

^{1}H NMR spectra were recorded at 25 °C on a Varian 600 MHz spectrometer. Mass spectra were recorded on a Bruker Autoflex III instrument. UV-vis absorption spectra were recorded on a Shimadzu UV-3100 spectrophotometer. The photoluminescence spectra were recorded on an Edinburgh FLS920 spectrofluorimeter under air at room temperature. FTIR spectra were obtained on a NICOLET iS50 instrument. Transmission electron microscopy (TEM) images were taken by a TECNAI F20 microscope. Diameter and diameter distribution of the nanoparticles were determined by a Malvern Zetasizer Nano instrument for dynamic light scattering (DLS). Confocal laser scanning microscopy (CLSM) images were taken using a LSM 800 Zeiss instrument, Germany.

***In Vitro* Photothermal Measurements.**

To investigate the photothermal performance of NPs, aqueous dispersions of different NPs (1 mL) were irradiated with a 635 nm laser (0.8 W cm^{-2}) for 5 min, and the temperature change was recorded by a thermocouple, and the photothermal images were acquired by an infrared thermal imager. In addition, the photothermal effect of the NPs was investigated using different laser power intensities (0.4, 0.6, 0.8 W and 1 W cm^{-2}) and the temperature change of different concentrations of NPs within 5 min was measured under the same power of laser irradiation. To study the NPs' photostability, five cycles of laser on/off were carried out.

Photothermal Conversion Efficiency of the NPs.

The photothermal conversion efficiency of the NPs was calculated according to a reported method.[1] First, the NPs (10^{-5} M) in aqueous solution were irradiated with a 635 nm laser (1 W cm^{-2}) for 300 s and then the laser was turned off. After about 800 s, the solution was cooled to room temperature. The photothermal conversion efficiency (η) was calculated according to the following equation

$$\eta=[hA(T_{Max}-T_{surr})-Q_{dis}]/I(1-10^{-A}) \quad (1)$$

where h and A respectively represent the heat transfer coefficient and the surface area of the container, T_{Max} and T_{sur}r represent the maximum temperature and the room temperature of the environment, Q_{dis} represents the heat dissipation of the solvent (water), I is the laser power employed (1 W cm^{-2}), and A is the absorbance of NPs at 635 nm. The value of hA is calculated from the following equation

$$\tau_s=m_Dc_D/hA \quad (2)$$

where τ_s is the time constant for heat transfer of the system, m_D and c_D are the mass and heat capacity, respectively, of the deionized water used to disperse the NPs. Q_{dis} represents the heat dissipation from the laser absorbed by the water, so Q_{dis} was calculated according to the following equation

$$Q_{dis}=m_D c_D(T_{Maxwater}-T_{surr})/\tau_{swater} \quad (3)$$

where $T_{max\ water}$ is the highest temperature of water. Based on the obtained data and Equation (1), the photothermal conversion efficiency of NPs was calculated.

Preparation of Reactive Oxygen and Nitrogen Species (RONS) Solutions.
The RONS solutions containing H_2O_2, TBHP and ClO^-, respectively, were purchased and diluted to the experimental concentration (200 μM) by 1×PBS (pH 7.4). In brief, •OH was produced by addition of ferrous chloride (0.1 M, 1 mL) into H_2O_2 solution (1.0 M, 1 mL) through a Fenton reaction. Accordingly, the concentration of •OH is the same as that of Fe^{2+} (50 mM). $O_2^{\bullet-}$ was generated from KO_2 (35.5 mg), which was directly added into dimethyl sulfoxide (10 mL) at a final concentration of 50 mM. $ONOO^-$ was prepared by addition of sodium hydroxide (1.5 M) into mixtures of sodium nitrite (0.6 M), hydrogen peroxide (0.7 M) and hydrochloric acid (0.6 M) at 0 °C, followed by purification through a short column of manganese dioxide to remove excess hydrogen peroxide. The concentration of $ONOO^-$ was determined by measurement of the absorption at 302 nm. $C[ONOO^-] = Abs_{302\ nm}/1.67$ (mM). $^1O_2$ was produced by addition of ClO^- solution (100 mM, 1 mL) into H_2O_2 solution (200 mM, 1 mL). According to this reaction, the concentration of $^1O_2$ is the same as that of ClO^- (50 mM). NO_2^- was generated from $NaNO_2$ (35 mg), which was directly added into deionized water (10 mL) at a final concentration of 50 mM.

Chemiluminescence and Fluorescence Imaging *In Vitro*.
For *in vitro* chemiluminescence imaging, including determinations of RONS selectivity, chemiluminescence spectra, and $ONOO^-$-activated sensitivity, etc., the concentration of Co2Ir NPs was 200 μg/mL. The chemiluminescence imaging was carried out with an Xenogen IVIS Lumina II system in a bioluminescent mode (exposure time 60 s) post RONS (200 μM) addition, unless otherwise specified. Tests were every 20 nm/step by the different filters. The environmental temperature for *in vitro* afterglow imaging was kept at 37 °C. The fluorescence spectra of Co2Ir NPs were measured by the same IVIS instrument in a fluorescent mode with excitation at 465 ± 10 nm (exposure time 1 s). The fluorescence and chemiluminescence images were analyzed by region-of-interest (ROI) analysis using the Living Image 4.2 Software.

FITC-Labeled Fibrin Clots Assay.
Fluorescein isothiocyanate (FITC)-labeled fibrin clots were induced by the addition of 10 U mL^{-1} thrombin and 2.5 mM $CaCl_2$ into a fibrinogen solution containing 1 mg mL^{-1} fibrinogen (200 μL) and 1 mg mL^{-1} FITC-labeled fibrinogen (20 μL), followed by incubation at 37 °C for 1 h. The clot was incubated with Co2Ir NPs (100 μg mL^{-1}, 500 μL) and further irradiated with a 635 nm laser (1 W cm^{-2}, 2 min) and ultrasound irradiation (1.0 W cm^{-2}, 1 MHz, 50% duty cycle) and evaluated through CLSM images.

***In vitro* hemolysis experiments.**
A hemolysis assay was conducted to evaluate the hemocompatibility of NPs. Primarily, the whole blood of a mouse treated with citrate was gathered and centrifuged at 3000 rpm for 3 min to obtain erythrocytes. Then, the erythrocytes were resuspended in an equal volume of PBS. PBS or water solution was used as negative and positive controls, respectively, which were added to 2 mL microtubes containing erythrocytes. Various concentrations (5, 10, 50, 100, 150 and 200 μg mL^{-1}) of NPs were incorporated into the erythrocytes for comparison with the controls. The mixture was then incubated in a 37 °C water bath for 2 h, followed by centrifugation at 3000 rpm for 10 min. After centrifugation, 100 μL of supernatant from each sample was transferred to a 96-well plate and the absorbance was recorded with an enzyme marker at 540 nm. The equation for calculation of hemolysis rate (%) is presented below. Hemolysis rate (%) = $(A_0 - A_1) \times 100\%/ (A_2 - A_1)$.

***In vitro* thrombolytic efficacy.**
The artificial thrombus was placed into a 5 mL glass vial, to which a mixture of 2.5 mL of PBS and 0.5 mL of different NPs solutions was added. The mixture was irradiated with a 635 nm laser and ultrasound (1.0 W cm^{-2}, 1 MHz, 50%). The weights of thrombus before and after thrombolytic treatment were measured to calculate the thrombolysis rate according to the equation: thrombolysis rate = (weight before treatment – weight after treatment)/weight before treatment.

***In Vitro* Cell Cytotoxicity Test.**
The human umbilical vein endothelial cells (HUVECs) were obtained from Peking Union Medical College Hospital (Peking, China). Fetal bovine serum (FBS), Ham's F12K, heparin, endothelial cell growth supplement (ECGS), L-glutamine, penicillin, and streptomycin were obtained from Corning (New York, USA). The cells were maintained in Ham's F12K medium with

heparin (0.1 mg mL^{-1}), ECGS (0.05 mg mL^{-1}), 10% FBS, 1% *L*-glutamine and 1% penicillin/streptomycin at 37 °C containing 5% CO_2. For *in vitro* cytotoxicity tests, HUVECs were seeded into 96-well plates with a density of 1 × 10^4 per well. After 24 h, the cells were incubated with Co2Ir NPs for another 24 h. The MTT assay was conducted following the standard protocol.

***In Vitro* Intracellular ROS Evaluation.**

The ROS were imaged using 2'7'-dichlorofluorescein diacetate (DCFH-DA). HUVECs (1 × 10^5 cells/well) were pretreated with four concentrations of NPs for 24 h, washed with PBS three times, and then were incubated with DCFH-DA (10 µM) for 30 min at 37 °C. Subsequently, residual agents were removed using PBS. The images were obtained using a confocal microscope (LSM 800 Zeiss, Germany). Each experiment was performed independently three times.

Cell migration assay

HUVECs in logarithmic growth phase with good growth status were taken and inoculated into cell culture 6-well plates at 5×10^5 cells/well, and cultured overnight at 37 °C in a 5% CO_2 incubator. Then the medium was removed and the cell monolayer was scratched along a straight line with a pipette tip. The cells were rinsed 3 times with PBS to remove the scratched cells. Photographs were taken at 0 h and then serum-free medium and a PBS solution of 2Ir NPs or Co2Ir NPs was added and incubated at 37 °C in a 5% CO_2 incubator. Photographs were taken and analyzed at 6 h, 12 h and 24 h, respectively.

Evaluation of angiogenesis *in vitro*

A tube formation assay was conducted to evaluate the angiogenic ability of HUVECs *in vitro* by cocultivation with blank, 2Ir NPs and Co2Ir NPs. Briefly, the medium was changed to serum-free Dulbecco's Modified Eagle Medium (DMEM)/F-12 (1:1, 1% penicillin–streptomycin), and the cells were starved for 24 h before the experiment. At the same time, the 2Ir NPs and Co2Ir NPs were immersed in DMEM/F-12 (1:1) at a ratio of 1:10 (v/v). The supernatant of the medium was collected 24 h later and filtered through a 0.22 mm filter membrane. The 15-well U-Slide angiogenesis plates,10 mL pipette tips, and 1 mL syringes were first precooled in a -80 °C freezer in advance, and the Matrigel was stored in a 4 °C freezer and melted for use. The precooled plates were placed flat on an ice box, and 10 mL of Matrigel was added to each well. Three replicate wells were set up for each group. Then, the plate was transferred to a cell incubator for 30 min until the Matrigel solidified. After the HUVECs were starved for 24 h, subjected to trypsin digestion, terminated, centrifuged, and the supernatant was discarded, the blank group was resuspended in serum-free DMEM/F-12, and the remaining groups were resuspended in their respective extract solutions. A 50 mL cell suspension was vertically seeded on Matrigel at a density of 1.5×10^4 cells/well. Then, the plates were incubated in an incubator until a large vascular network was observed to form under an optical microscope. The medium in each well was aspirated, and the wells were washed with PBS. Images were taken of the specimens as tubes under a microscope. The results were analyzed by ImageJ software.

Construction of thrombus model

All animal procedures were performed in accordance with the Guidelines for Care and Use of Laboratory Animals approved by the Animal Ethics Committee of the China Technology Industry Holdings (Shenzhen) Co., Ltd. Male C57 BL/6J mice (8-10 weeks old) were anesthetized using isoflurane (2% of isoflurane, 0.5 mL min^{-1} oxygen) and the neck hair was shaved off. Then the skin around the neck was cut with surgical scissors, and the connective tissue and fat were peeled away to expose the carotid vessels. A 10% aqueous solution of $FeCl_3$-soaked filter paper (2 × 1 mm) was placed on the surface of the exposed carotid artery for 10 min, then the filter paper was removed. The vessel and surrounding tissue were washed with sterilized saline solution. An aggregated embolus could be observed under a somatic microscope. A laser speckle imaging system (RWD, RFLSI III, Shenzhen, China) was used to monitor the hemodynamic changes before and after the induction of carotid artery thrombus using $FeCl_3$. All animal procedures were approved by the China Technology Industry Holdings (Shenzhen) Co., Ltd.

***In vivo* chemiluminescence imaging of thrombus**

The C57 BL/6J mice (8-10 weeks old) with a thrombus model were intravenously treated with PBS or Co2Ir NPs (5 mg kg^{-1}), respectively. The mice were then anesthetized using isoflurane (2% of isoflurane, 0.5 mL min^{-1} oxygen) and placed on a heating platform at 37 °C. At predetermined times (0, 30, 60, 90, 120, 180, and 240 min) post-NPs administration, the *in vivo* chemiluminescence imaging was carried out using an IVIS imaging system.

Evaluation of the thrombolytic effect of different formulations *in vivo*

To evaluate the therapeutic effect of different formulations on thrombi, the mice with carotid thrombosis or with lower extremity arterial thrombosis were randomly divided into three groups (n = 5 mice): PBS, free UK or Co2Ir NPs groups. The different formulations were intravenously injected into the mice via the tail vein. For the NIR light-treated groups, the carotid arteries of the mice were illuminated with a 635 nm laser (1.0 W cm^{-2}) and ultrasound irradiation (1.0 W cm$^{-2,}$ 1 MHz, 50% duty cycle) (5 min per session) for 120 min at 60 min post-NP administration. The temperature changes at the irradiation sites were monitored using an infrared thermal imaging system (Guide, Zhejiang, China). At different times (10, 20, 50, 60, 90, and 120 min) after the treatments, a laser speckle imaging system was used to observe and record the blood flow velocity

and blood flow at thrombosis sites. Finally, the mice were euthanized, and the carotid arteries were excised for section and H&E staining. The areas and morphology of the thrombi after different treatments were observed under an optical microscope. The sections were analyzed using the Image J2x software (version 2.1.4.7) and thrombolytic efficiency was determined by the area ratio of vascular occlusion to total vascular lumen.

***In vivo* biosafety evaluation.**
To test the potential toxicity of Co2Ir NPs, healthy C57BL/6 mice (8-10 weeks old) were intravenously injected with PBS or Co2Ir NPs (5 mg kg^{-1}). One week after PBS or NPs administration, the mice were euthanized, and blood was drawn. Then the hematological parameters, including white blood cell count, red blood cell count, hemoglobin concentration and platelet crit were analyzed using an automated hematology analyzer.

Statistical analysis
Data are denoted as the mean ± standard deviation (SD). The significance between experimental and control groups was determined by unpaired 2-tailed Student's t-test using the GraphPad Prism 7. A value of $p < 0.05$ was considered statistically significant. * $p < 0.05$, ** $p < 0.01$, *** $p < 0.001$.

Synthesis

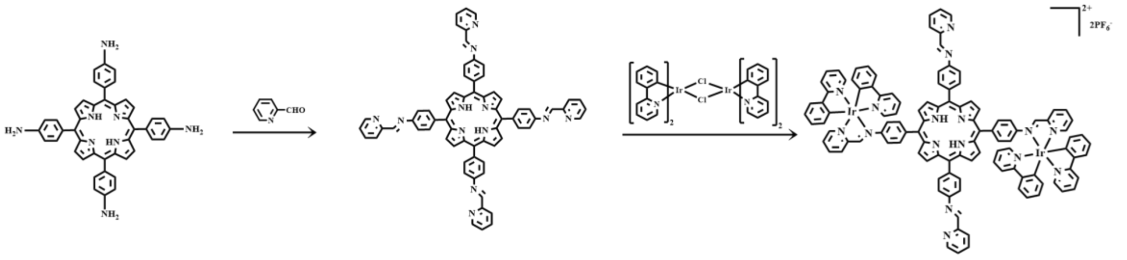

TPP-4NH$_2$ L4 2Ir-NN

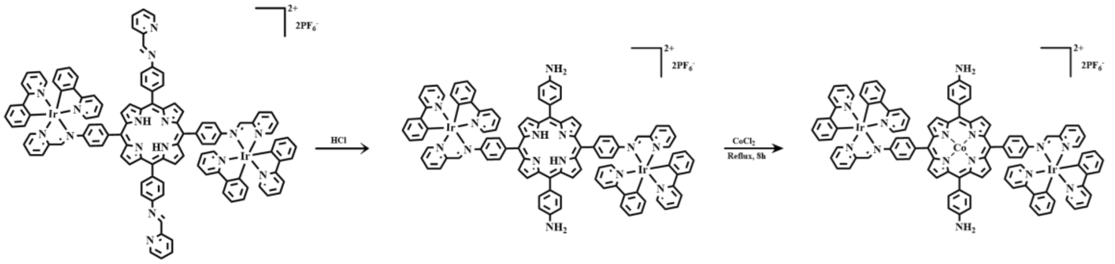

2Ir-NN 2Ir Co2Ir

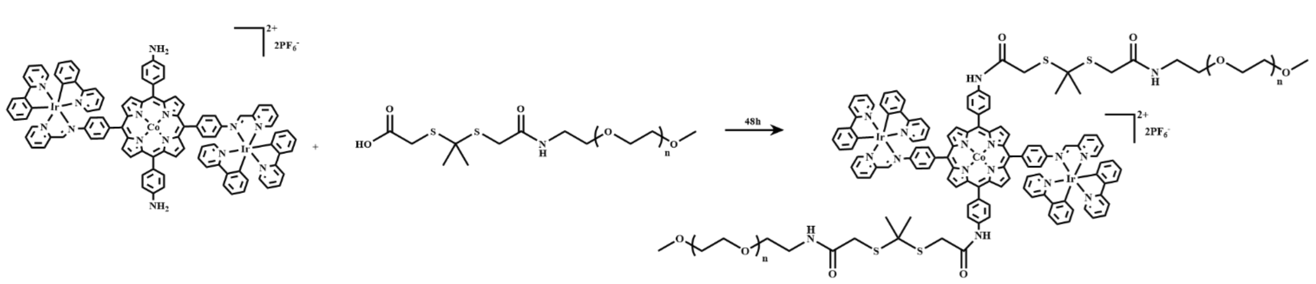

Co2Ir Co2Ir NPs

Scheme S1. The synthesis of Co2Ir NPs.

Synthesis.
L4 was synthesized as reported previously.[2]
Synthesis of 2Ir. $[Ir(ppy)_2Cl]_2$ (0.107 g, 0.1 mmol) and auxiliary ligand L4 (0.1030 g, 0.1 mmol) were dissolved in a mixture of methanol (30 mL) and dichloromethane (30 mL), and the solution was refluxed for 6 h under the protection of N_2. After cooling to room temperature, excess KPF_6 was added to the mixture (to replace counterion Cl^-). After stirring for 0.5 h,

filtering removed the excess potassium salts, and then rotary evaporation removed solvent to give the crude product 2Ir-NN. No purification was required. The crude product was directly used for the next reaction.

2Ir-NN (0.2032 g, 0.1 mmol) was dissolved in 1 mL of acetone, then 20 mL of concentrated hydrochloric acid was added to the solution which was stirred at room temperature for 2 hours. The mixture was then extracted with dichloromethane and water to obtain 2Ir, with a yield of 80%. Molecular formula $C_{100}H_{72}Ir_2N_{14}$, and relative molecular mass of 1854.53 g/mol; found $[M]^{2+}$ = 927.1587 g/mol. ^{1}H NMR (500 MHz, DMSO-d_6) δ 9.99 (s, 1H), 9.07 (s, 1H), 8.87 (s, 1H), 8.66 (d, *J* = 51.7 Hz, 3H), 8.41 (t, *J* = 37.6 Hz, 5H), 8.12 – 7.81 (m, 12H), 7.46 (d, *J* = 6.1 Hz, 1H), 7.39 (d, *J* = 7.0 Hz, 1H), 7.24 (s, 3H), 7.13 – 7.03 (m, 3H), 6.98 (d, *J* = 7.3 Hz, 2H), 6.32 (d, *J* = 8.0 Hz, 2H).

Synthesis of Co2Ir. 2Ir (0.1 mmol, 0.1854.53 g) and $CoCl_2$ (0.5 mmol, 0.064435 g) were added to a 50 mL burette with DMF (30 mL) as the reaction solvent, and the mixture was stirred at 160 °C for 8 h. The product was extracted with dichloromethane and water, and the solvent was removed by rotary evaporation, and the red solid product, i.e., Co2Ir, was dried and obtained in a yield of 85%: Molecular formula $C_{100}H_{70}CoIr_2N_{14}$, and relative molecular mass of 1911.45 g/mol; found $[M]^{2+}$ = 955.67 g/mol.

Synthesis of Co2Ir NPs. Co2Ir (0.1 mmol, 0.1914 g) and MPEG2000-TK-COOH (0.2 mmol, 0.4035 g) was dissolved in 5 ml of DMSO, then 1-ethyl-3-(3-dimethylaminopropyl)carbodiimide (EDC) (0.1 mmol, 0.01917 g) and *N*-hydroxysuccinimide (NHS) (0.1 mmol, 0.0115 g) were added and the mixture was stirred at room temperature for 2 hours. Next, Co2Ir was dissolved in 1 ml of DMSO and added drop by drop. The mixture was stirred in the dark for 48 hours. After the reaction was completed, the liquid was taken out and dialyzed to obtain water-soluble Co2Ir NPs.

Synthesis of 2Ir NPs. 2Ir NPs were prepared using the same method as described above using 2Ir instead of Co2Ir.

NMR and MS spectra of compounds

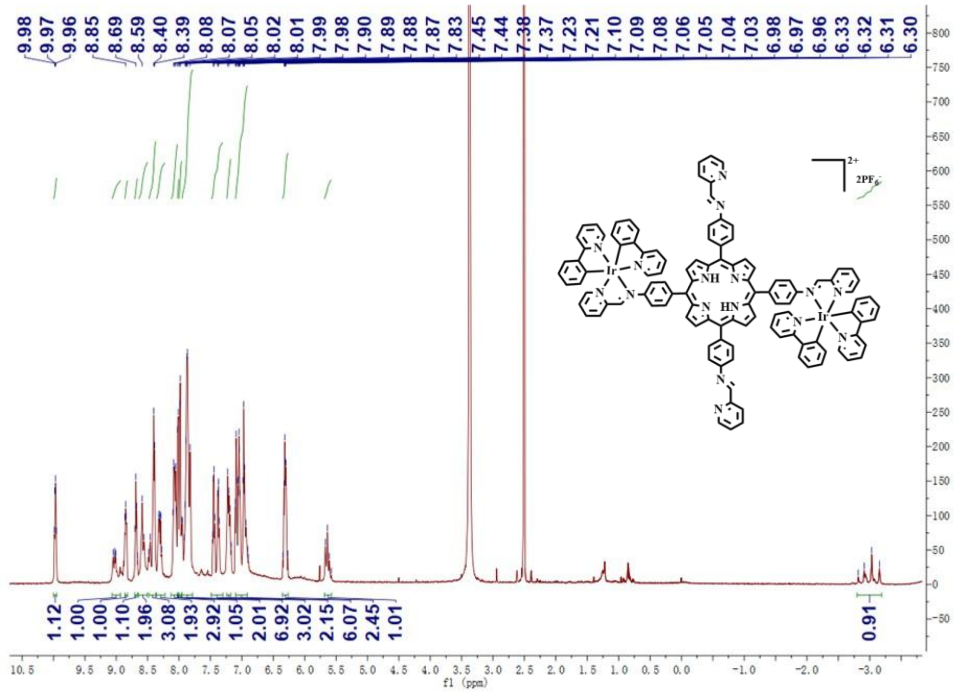

Figure S1. ^{1}H NMR spectrum of 2Ir-NN in DMSO-d_6 at room temperature.

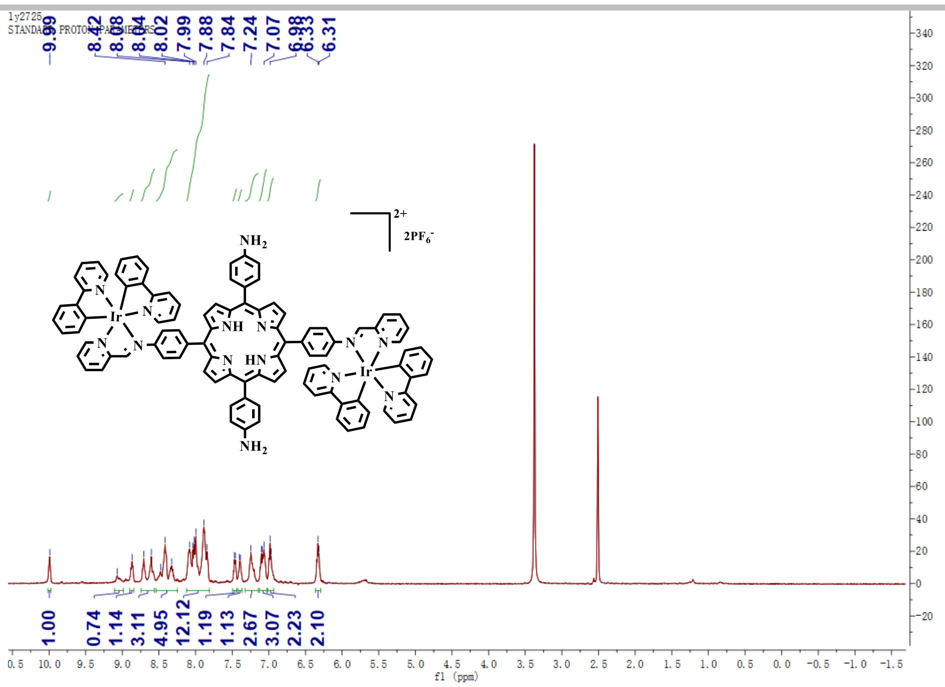

Figure S2. ^{1}H NMR spectrum of 2Ir in DMSO-d_6 at room temperature.

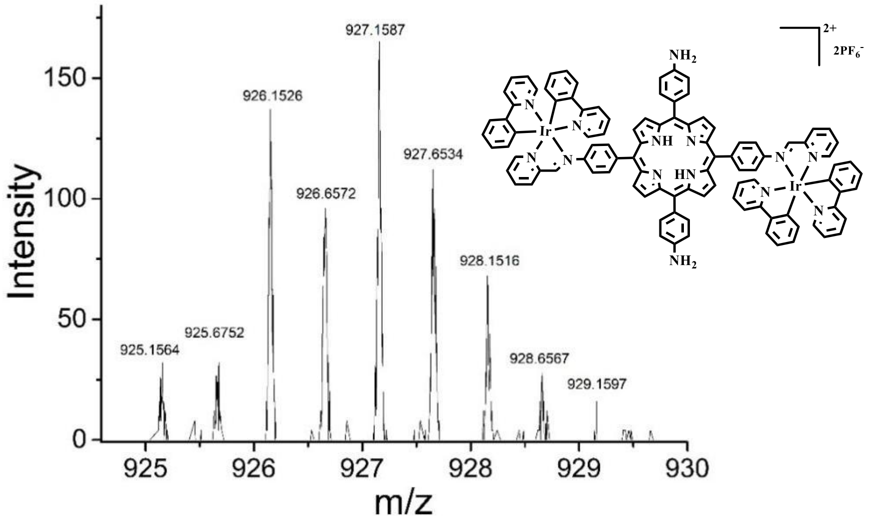

Figure S3. Mass spectrum of 2Ir at room temperature.

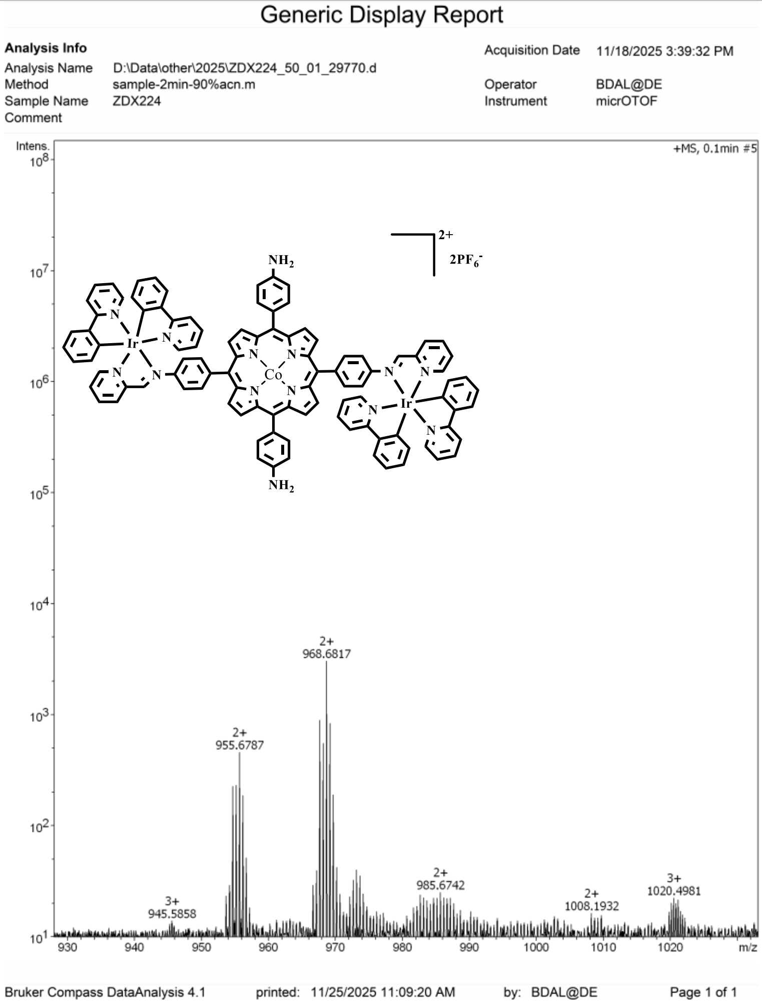

Figure S4. Mass spectrum of Co2Ir at room temperature.

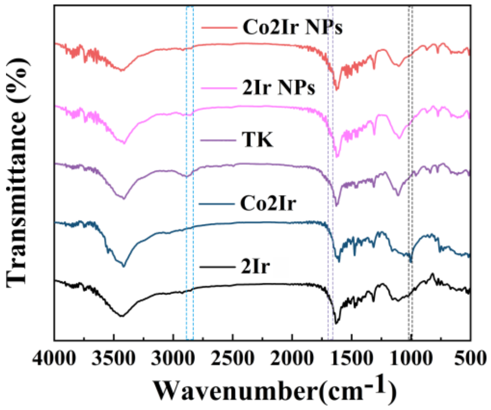

Figure S5. FTIR spectra of 2Ir, Co2Ir, TK (MPEG2000-TK-COOH) 2Ir NPs and Co2Ir NPs.

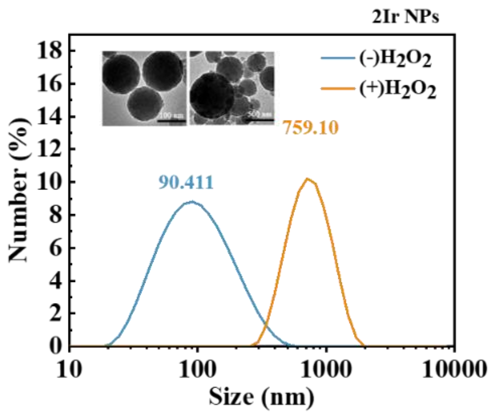

Figure S6. Size and TEM image distribution of 2Ir NPs before (-) and after (+) treatment with H_2O_2 by DLS.

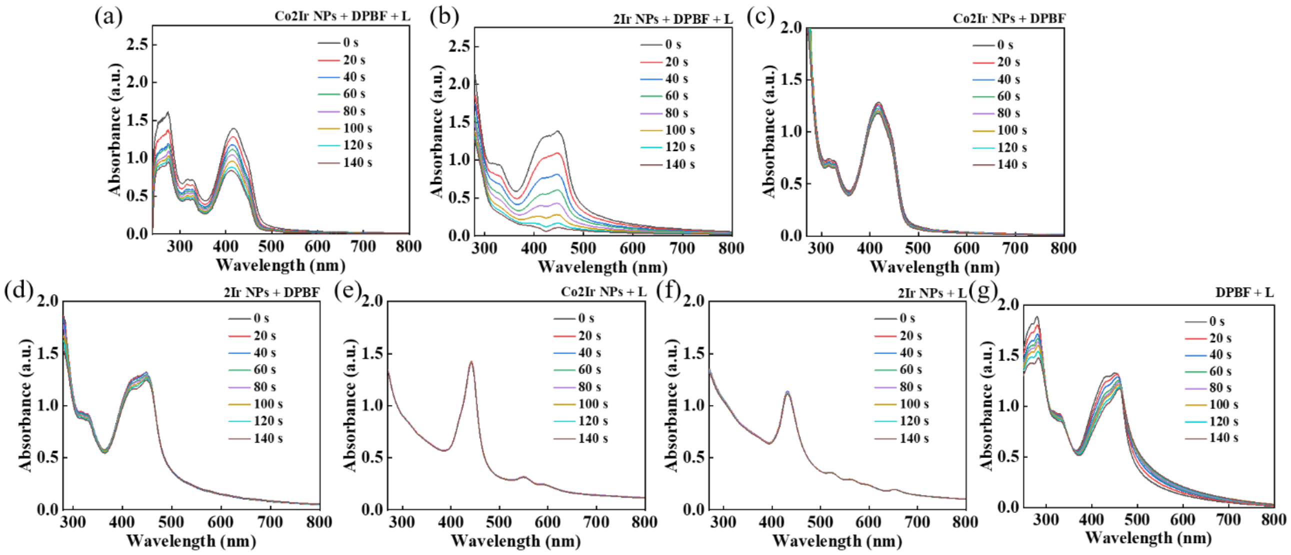

Figure S7. UV–vis absorption spectral changes of DPBF (1×10^{-5} M) in the presence of Co2Ir NPs (a) and 2Ir NPs (b) upon exposure to light (635 nm, 0.8 W cm^{-2}) during 140 s. UV–vis absorption spectral changes of DPBF (1×10^{-5} M) in the presence of Co2Ir NPs (c) and 2Ir NPs (d) upon exposure to light (635 nm, 0.8 W cm^{-2}) during 140 s. UV–vis absorption spectral changes of Co2Ir NPs (e) and 2Ir NPs (f) upon exposure to light (635 nm, 0.8 W cm^{-2}) during 140 s. UV–vis absorption spectral changes of DPBF (1×10^{-5} M) upon exposure to light (635 nm, 0.8 W cm^{-2}) during 140 s.

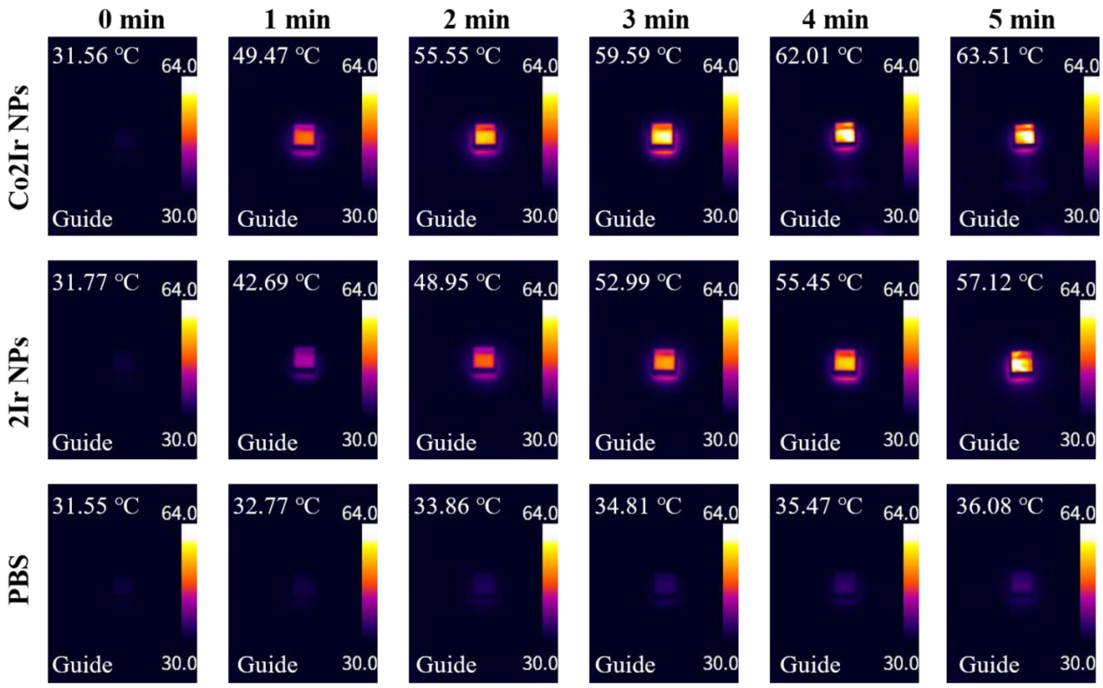

Figure S8. Infrared thermal images of Co2Ir NPs, 2Ir NPs and PBS under laser irradiation.

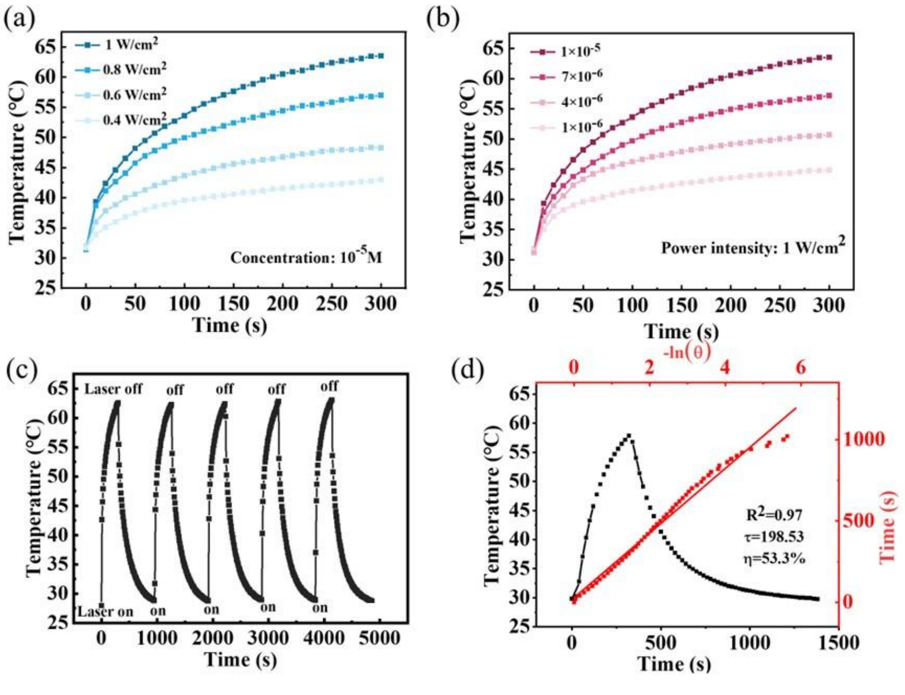

Figure S9. (a) Laser power density and (b) concentration dependence of the photothermal effects of Co2Ir NPs under 635 nm laser irradiation. (c) Temperature variations of Co2Ir NPs (10^{-5} M) under irradiation at a power density of 1 W cm^{-2} for five light on/off cycles. (d) Plot of cooling time versus negative natural logarithm of the temperature obtained from the cooling stage of 2Ir NPs.

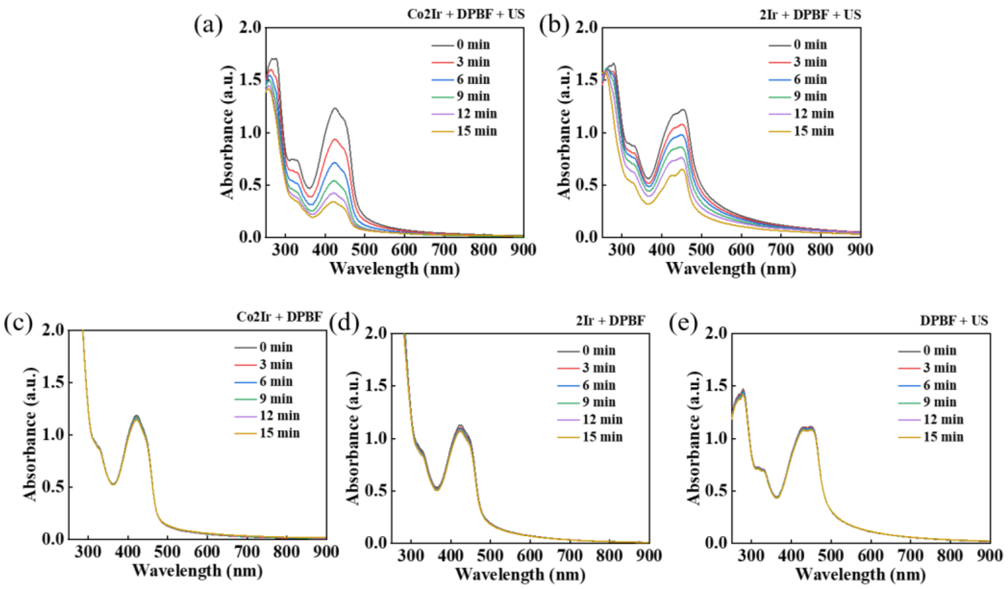

Figure S10. UV–vis absorption spectra of DPBF (1×10^{-5} M) in the presence of Co2Ir NPs (a), and 2Ir NPs (b) under ultrasound irradiation (1.0 W cm^{-2}, 1 MHz, 50% duty cycle). (c) UV–vis absorption spectra of DPBF in the presence of Co2Ir NPs without ultrasound irradiation; (d) UV–vis absorption spectra of DPBF in the presence of 2Ir NPs without ultrasound irradiation; (e) UV–vis absorption spectra of DPBF under ultrasound irradiation (1.0 W cm^{-2}, 1 MHz, 50% duty cycle).

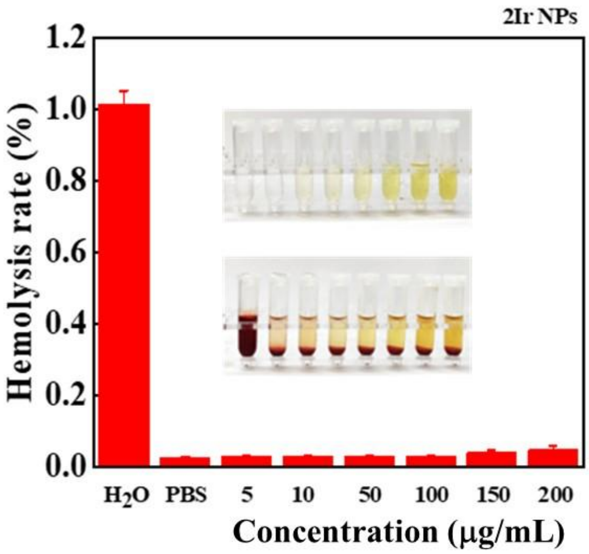

Figure S11. The hemolysis rate of red blood cells treated with water and different concentrations (μg/mL) of 2Ir NPs. Inset shows samples in the presence or absence of the solution of red blood cells upon different treatments.

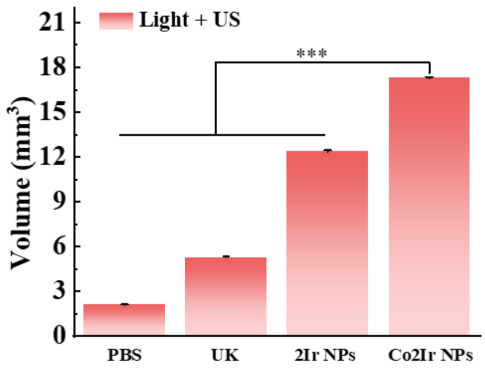

Figure S12. Quantitative analysis of the volume of thrombolysis under light exposure and US (corresponding to Figure 3e).

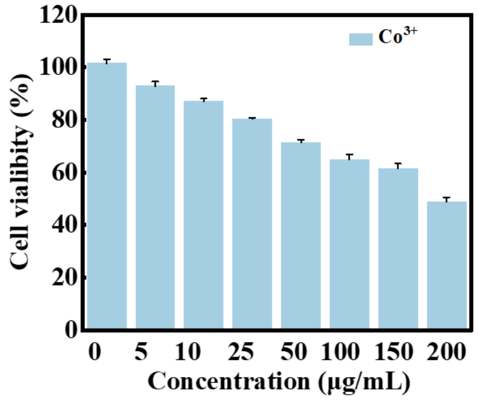

Figure S13. Cell viability was measured by MTT assay after HUVEC cells were treated with different concentrations of $CoCl_3$ for 24 h.

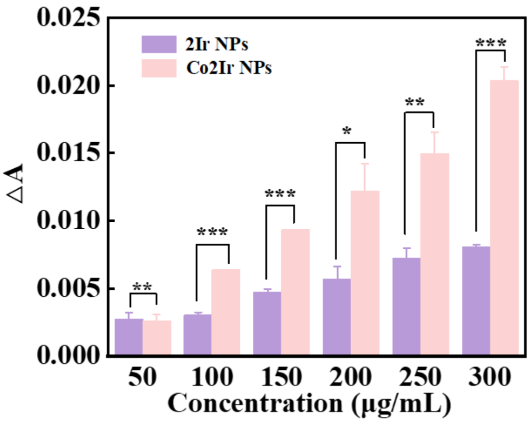

Figure S14. ROS scavenging ability of Co2Ir NPs and 2Ir NPs. Data are presented as mean ± SD (n = 3). $p < 0.05$ was considered statistically significant. *$p < 0.05$, **$p < 0.01$, ***$p < 0.001$, ****$p < 0.0001$.

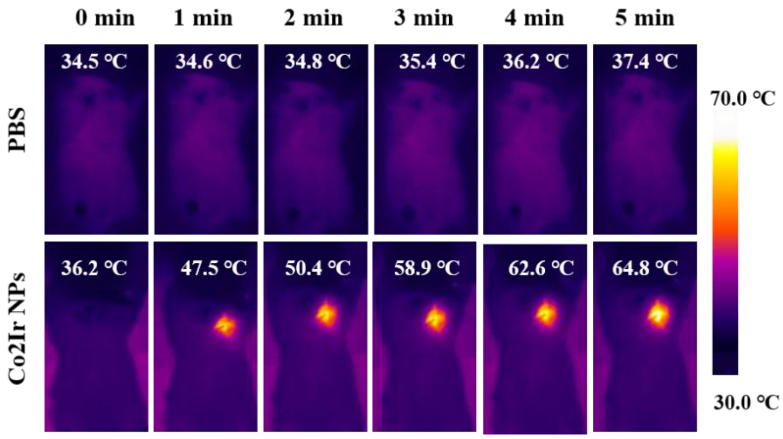

Figure S15. Thermal images of the thrombus site in mice intravenously injected with PBS or Co2Ir NPs (5 mg kg^{-1}) after 635 nm laser irradiation (0.8 W cm^{-2}) for different times.

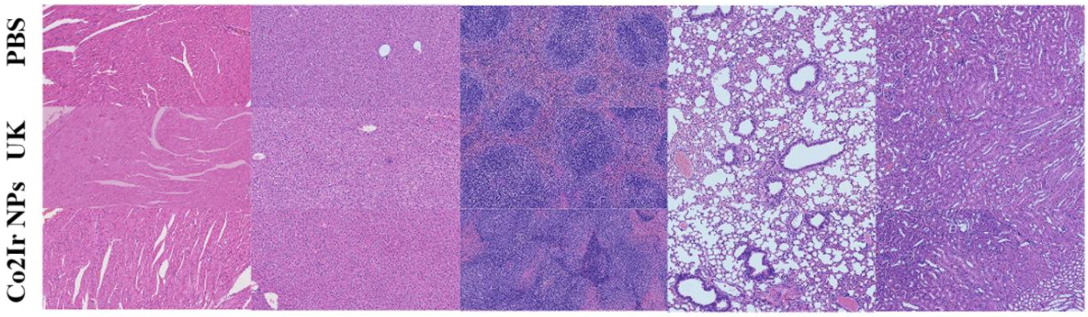

Figure S16. H&E staining of tissue sections from major organs of mice which were euthanized after injection with PBS, free UK and Co2Ir NPs. Left to right: brain, heart, liver, spleen, lung, and kidney.

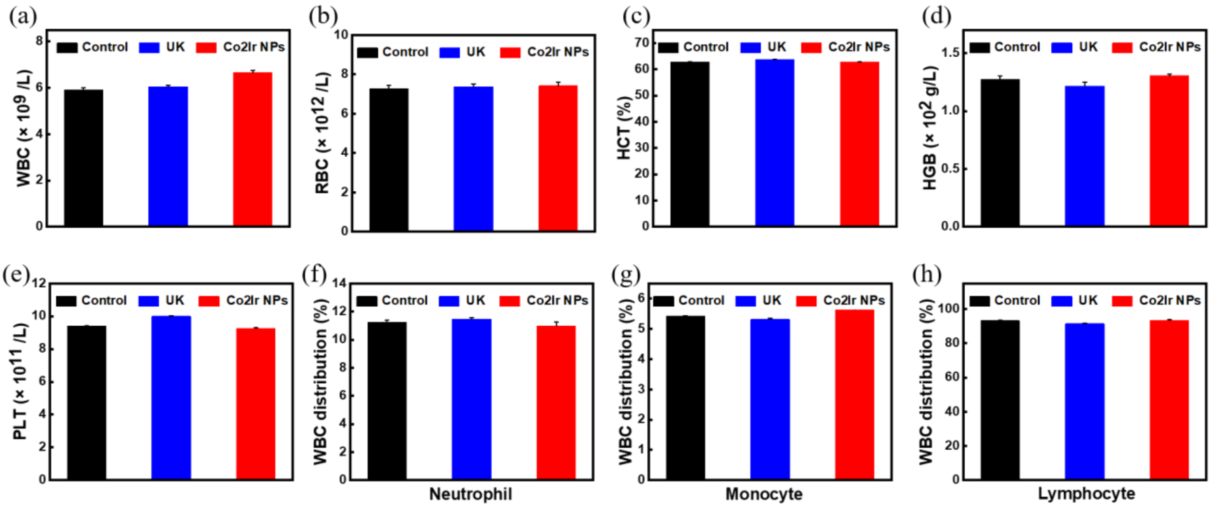

Figure S17. The routine blood test data of the mice with different treatments. Data are presented as mean ± SD (n = 3 mice). WBC is white bold cell; RBC is red blood cell; HCT is hematocrit; HGB is hemoglobin; PLT is platelet count.

1. X. Liu, B. Li, F. Fu, K. Xu, R. Zou, Q. Wang, B. Zhang, Z. Chen and J. Hu, *Dalton Trans.*, 2014, **43**, 11709-11715.
2. Z. W. Wang, L. J. Li, W. J. Wang, R. L. Wang, G. Z. Li, H. Bian, D. X. Zhu and M. R. Bryce, *Dalton Trans.,* 2023, **52**, 1595-1601.
